# Antibiotic Susceptibility Patterns, Biofilm Formation and *esp* Gene among Clinical Enterococci: Is There Any Association?

**DOI:** 10.3390/ijerph16183439

**Published:** 2019-09-17

**Authors:** Poh Leng Weng, Ramliza Ramli, Rukman Awang Hamat

**Affiliations:** 1 Department of Medical Microbiology and Parasitology, Faculty of Medicine and Health Sciences, Universiti Putra Malaysia, Serdang, Selangor 43400, Malaysia; fievel_w@yahoo.com; 2 Department of Medical Microbiology and Immunology, Universiti Kebangsaan Malaysia Medical Centre, Jalan Yaakob Latif, Bandar Tun Razak, Kuala Lumpur 56000, Malaysia; ramliza@ppukm.ukm.edu.my

**Keywords:** *esp* gene, biofilm, antibiotic resistance, enterococci

## Abstract

Enterococci are commonly found in humans, animals and environments. Their highly adaptive mechanisms are related to several virulent determinants and their ability to resist antibiotics. Data on the relationship between the *esp* gene, biofilm formation and antibiotic susceptibility profiles may differ between countries. This cross-sectional study was conducted to determine the proportion of *esp* gene and biofilm formation among *Enterococcus faecalis* and *Enterococcus faecium* clinical isolates. We also investigated the possible association between the *esp* gene with antibiotic susceptibility patterns and biofilm formation. The isolates were collected from clinical samples and identified using biochemical tests and 16SRNA. Antibiotic susceptibility patterns and a biofilm assay were conducted according to the established guidelines. Molecular detection by PCR was used to identify the *esp* gene using established primers. In total, 52 and 28 of *E. faecalis* and *E. faecium* were identified, respectively. *E. faecium* exhibited higher resistance rates compared to *E. faecalis* as follows: piperacillin/tazobactam (100% versus 1.9%), ampicillin (92.8% versus 1.9%), high-level gentamicin resistance (HLGR) (89.3% versus 25.0%) and penicillin (82.1% versus 7.7%). *E. faecium* produced more biofilms than *E. faecalis* (59.3% versus 49.0%). *E. faecium* acquired the *esp* gene more frequently than *E. faecalis* (78.6% versus 46.2%). Interestingly, the associations between ampicillin and tazobactam/piperacillin resistance with the *esp* gene were statistically significant (*X*^2^ = 4.581, *p* = 0.027; and *X*^2^ = 6.276, *p* = 0.012, respectively). Our results demonstrate that *E. faecium* exhibits high rates of antimicrobial resistance, *esp* gene acquisition and biofilm formation. These peculiar traits of *E. faecium* may have implications for the management of enterococcal infections in hospitals. Thus, concerted efforts by all parties in establishing appropriate treatment and effective control measures are warranted in future.

## 1. Introduction

Human enterococci are normal commensals that reside in the gastrointestinal system as part of our gut microbiota. Surprisingly, they have gained greater interest among scientists owing to their ability to emerge as one of the most important nosocomial opportunistic pathogens worldwide. These highly versatile bacteria are responsible for causing persistently high mortality and morbidity rates in patients with bacteremia, surgical site infections and urinary tract infections [1,2]. Notably, the crude mortality rates of 13% to 68% have been reported for enterococcal bacteremia in several retrospective studies [3,4]. Among the predominant enterococci observed in humans, *Enterococcus faecalis* and *Enterococcus faecium* are the most significant pathogens causing hospital-acquired infections [5]. An initial wave of enterococcal infections was dominated by *E. faecalis*, which contributed to 90% of the overall cases, while the remaining 10% were due to *E. faecium* [6,7]. However, this was replaced by *E. faecium* as a result of its remarkable resistance to several antibiotics which include vancomycin, ampicillin and high-level aminoglycoside-resistance (HLAR) [8]. Enterococci are well known to demonstrate a high degree of adaptability towards harsh environments, as they can endure extreme temperatures (from 10 °C to >45 °C), as well as high salt content and pH levels [6].

In hospital settings, enterococcal survival derives from their ability to rapidly adapt to antimicrobial exposures [9]. Their ability to exhibit rapid genetic mutations has created substantial threats to the swift development of multi-drug resistance strains globally. Vancomycin-resistant enterococci (VRE), for instance, are now being identified as the fourth and fifth leading pathogens causing sepsis in North America and Europe, respectively [10]. This is mainly due to their rapid acquisition of mobile genetic elements (MGEs) such as plasmids, conjugative transposons, integrons and pathogenicity islands (PAIs) [11]. Several antibiotic and virulent determinants have been found in these mobilomes which are readily transmissible [12].

In addition, biofilm formation is one of the strategies for the enterococci to evade the host’s immune response and the inhibitory or killing effects of antibiotics [13,14]. This self-produced extracellular matrix also provides a suitable microenvironment for enterococci to grow and facilitates the transmission of mobile genetic elements (MGEs) between bacteria [15]. Enterococcal biofilms have been implicated in indwelling device-related infections such as prosthetic valve endocarditis, prosthetic joint infections and catheter-related infections [16]. Several virulence determinants such as enterococcal surface protein (*esp*) gene, gelatinase (*gelE*) gene and others have been shown to be involved in the propensity of enterococci to form biofilms [14,17]. Nonetheless, many reports have supported the contribution of virulence genes in the formation of enterococcal biofilms, whereas some researchers have reported conflicting findings [13,17,18]. Furthermore, the association of the *esp* gene with biofilm formation displays considerable variations between *E. faecalis* and *E. faecium* [17,19,20]. With the conflicting reports on the traits of these highly adaptive bacteria, there remains a question of to what extent biofilm production, virulence and resistance among enterococci are associated. We believe the findings could offer an insight into supporting the proper management of enterococcal infections in hospitals.

Thus, this prompted us to determine the prevalence of the *esp* gene and to screen for biofilm producers among *E. faecalis* and *E. faecium* clinical isolates. Our study was also intended to determine the association of the *esp* gene with antibiotic susceptibility patterns and biofilm formation. To the best of our knowledge, existing data on the association between these variables in Malaysian enterococcal isolates is still lacking. We have previously established the wide distribution of virulent genes among our clinical enterococci, excepting the *esp* determinant and biofilm formation [21]. 

## 2. Materials and Methods

### 2.1. Clinical Enterococci Isolation

Clinical enterococci were isolated from a tertiary hospital during a one-year study period (from 1 December 2014 to 1 December 2015) using a cross-sectional study approach. A total of 52 and 28 *E. faecalis* and *E. faecium* isolates, respectively, were obtained from various samples such as pus (*n* = 41), blood (*n* = 27), urine (*n* = 8), high vaginal swabs (*n* = 2) and others (*n* = 2). All isolates were cultured in several media accordingly (selective and non-selective) based on established protocols. The identity of the isolates was confirmed by using several procedures such as conventional biochemical tests [22], Remel RapID Strep Kit (Oxford, UK), and species-specific PCR which has previously been described by Kariyama et al. [23].

### 2.2. Antibiotic Susceptibility Testing

The Kirby–Bauer disk diffusion method was used for antibiotic susceptibility testing. The following antibiotic discs were used: penicillin (10 Unit, Oxoid), ampicillin (10 µg, Oxoid) high-level gentamicin resistance (HLGR) (120 µg, Oxoid), piperacillin/tazobactam (100/10 μg, Oxoid), teicoplanin (30 µg, Oxoid) and vancomycin (30 µg, Oxoid). All results were interpreted according to the Clinical and Laboratory Standards Institute guidelines [24].

### 2.3. Screening of Enterococcal Biofilms

The biofilm formation was screened by using the colorimetric assay according to the established protocol with slight modifications [13]. Briefly, a total of 200 μL bacterial solution containing trypticase soy broth (TSB) (Oxoid, UK) supplemented with 0.25% glucose was added into a sterile 96-well microtiter plate. The microtiter plate was incubated at 37 °C for 24 h. Then, the medium was gently removed and the wells were washed three times with 100 μL of Phosphate Buffer Saline (PBS) before being allowed to dry for 1 h. The wells were stained with 100 μL of 0.2% crystal violet (Merck, Germany) for 15 min at room temperature. The unbound crystal violet was removed by washing the wells three times with PBS and the crystal violet was solubilized by adding 200 μL of 33% acetic acid. Finally, the plate was read at 595 nm using a microtiter plate reader (Dynex Technologies, Chantilly, VA, USA). The procedure was performed in triplicate for each sample. The strains were classified as no biofilm producer, weak biofilm producer, moderate biofilm producer and strong biofilm producer, according to the established protocol by Stepanovic et al. [25].

### 2.4. Molecular Detection of the Enterococcal Surface Protein (esp) Gene

DNA extraction was performed by using a Masterpure Complete DNA purification kit (Epicentre Technologies, Wisconsin, WI, USA) according to the manufacturer’s instructions. The *esp* gene was detected using PCR with established forward and reverse primers as follows: *esp* F, 5′-TTG CTA ATG CTA GTC CAC GAC C-3′; *esp* R, 5′-GCG TCA ACA CTT GCA TTG CCG AA-3′ (Ramadhan and Hegedus). The reaction mixtures were prepared which consisted of the following: 25 µL PCR master mixture of 12.5 µL Gotaq green master mix (Promega, USA), 0.5 µL primer (10 mM), 9.5 µL sterile ultrapure water (Milipore, Burlington, MA, USA), and 2 µL DNA. The PCR conditions were carried out as follows: an initial denaturation step at 95 °C for 15 min; 30 cycles of denaturation at 90 °C for 30 s, annealing at 58 °C for 1 min, and an extension at 72 °C for 1 min; a final extension at 72 °C for 10 min. The PCR products were viewed under the UV imager (Alpha Imager™ 2200, Alpha Innotech Corporation, California, CA, USA) using 0.8% agarose gel at 70 V with Lambda *Hind*III marker.

### 2.5. Statistical Analysis

Chi-square analysis was employed to test the relationship between certain variables, such as the *esp* gene versus antibiotic susceptibility patterns and biofilm formation. The *p* value of <0.05 was considered as statistically significant. The analysis was conducted using (SPSS Inc., v. 19, Chicago, IL, USA).

## 3. Results

### 3.1. Antibiotic Susceptibility Patterns

Of 80 clinical specimens, 63.8% and 36.2% were from invasive and non-invasive isolation sites, respectively. *E. faecalis* isolates exhibited high susceptibility rates towards ampicillin (98.1%), piperacillin/tazobactam (98.1%), penicillin (92.3%), and high-level gentamicin resistance (HLGR) (75.0%). On the other hand, *E. faecium* isolates demonstrated high resistance rates towards piperacillin/tazobactam (100%), ampicillin (92.8%), HLGR (89.3%) and penicillin (82.1%). However, no resistance to teicoplanin or vancomycin was detected in either species (Table 1).

### 3.2. Biofilm Colometric Assay

In total, 41 (52.6%) clinical enterococci produced biofilms whereas 37 (47.4%) did not. Among biofilm producers, *E. faecium* isolates exhibited higher biofilm formation than *E. faecalis* (59.3% versus 49.0%). However, strong biofilm producers were demonstrated by both species in almost equal percentages (28.0% and 25.0% of *E. faecalis* and *E. faecium*, respectively). The distribution of biofilm formation according to the classification of biofilm formation among *E. faecalis* and *E. faecium* isolates is shown in Figure S1 in Supplementary Materials. Only 78 clinical enterococci were recruited as two isolates were lost during the procedure (one each for *E. faecalis* and *E. faecium*).

### 3.3. Detection of the esp Gene

In our study, the overall prevalence of the *esp* gene among clinical enterococci isolates was 57.5%. According to the type of enterococcal species, 78.6% and 46.2% of *E. faecium* and *E. faecalis* carried the *esp* gene, respectively. PCR products were visualized for the presence of the *esp* gene (Figure S2 in Supplementary Materials).

### 3.4. Associaton between the esp Gene with Antimicrobial Resistance and Biofilms

The association between each antibiotic resistance and the presence of the *esp* gene is shown in Table 2. The *esp* gene was present in ampicillin-resistant isolates more frequently than in ampicillin-sensitive isolates (25.0% versus 8.7%). Similarly, tazobactam/piperacillin-resistant isolates exhibited a higher frequency of *esp* gene compared to tazobactam/piperacillin-sensitive isolates (25.0% versus 10.0%). The associations between ampicillin and tazobactam/piperacillin resistance with the *esp* gene were statistically significant (*X*^2^ = 4.581; *p* = 0.032 and *X*^2^ = 6.276; *p* = 0.012, respectively). There were no significant associations found between penicillin resistance and HLGR with the *esp* gene (*X*^2^ = 0.498; *p* = 0.481 and *X*^2^ = 2.035; *p* = 0.154, respectively). In addition, there was no significant association between the presence of the *esp* gene and biofilm formation (*X*^2^ = 0.007; *p* = 0.934). Table 3 shows the association between the *esp* gene and biofilm formation among our clinical enterococci isolates.

## 4. Discussion

In general, *E. faecium* exhibited higher resistance rates toward selected antibiotics than *E. faecalis* in our study. Ampicillin and HLGR are of concern. Ampicillin-resistant *E. faecium* isolates have now become widespread worldwide [26,27]. The reduced affinity of penicillin-binding proteins and plasmid-mediated β-lactamases is responsible for these resistance traits [28]. It is believed that the dissemination of the chromosomally encoded penicillin-binding protein 5 (*PBP5*) gene by enterococcal conjugative plasmids among clinical enterococci isolates might contribute to a global spread [29]. Nonetheless, the synergistic combination of penicillin/ampicillin (β-lactams drugs) and aminoglycoside can be useful in treating serious enterococcal infections [30]. In our study, *E. faecium* exhibited higher rates of HGLR than *E. faecalis* isolates (89.3% versus 25.0%), thus this synergism could not be the best therapeutic option in managing infections caused by *E. faecium* in our clinical setting. Our findings are consistent with data from other countries as well [31,32].

One of the novel strategies the enterococci use in order to be consistently present in hospital environments is the formation of biofilms which could allow them to adhere to any surfaces [33]. It has been proposed that biofilms are pivotal in the pathogenesis of enterococcal infections, especially in urinary tract infections [34]. In the present study, a higher production of biofilms was observed among *E. faecium* than *E. faecalis* isolates (59.3% versus 49.0%). Our finding is in accordance with other studies in which *E. faecium* clinical strains produced more biofilms than others [35]. Higher rates of biofilm formation in *E. faecium* were also observed in Poland and Spain (77.8 and 75%, respectively) [15,36]. However, several studies have found a reduced formation of biofilms among *E. faecium* compared to *E. faecalis* isolates [12,18,27]. For instance, biofilm formation was more highly reported in *E. faecalis* than *E. faecium* isolates in Italy (80% versus 48%) [37]. In the UK, among 109 enterococci, 100% and 42% of *E. faecalis* and *E. faecium* isolates produced biofilms, respectively [38]. Surprisingly, none of the 25 *E. faecium* produced biofilms compared to 26% of *E. faecalis* isolates in a study among 171 enterococci in India [39]. It seems that there are differences in terms of the prevalence rates of biofilm formation among these two important clinical enterococci. Nonetheless, the true prevalence of biofilm formation among clinical enterococci is still unknown and it may vary according to the type of methodology, nature of specimens, type of strains and geographical location. 

In our study, *E. faecium* carried the *esp* gene more frequently than *E. faecalis* (78.6% versus 46.2%). The prevalence rate of the *esp* gene among these two common enterococci species varies from country to country. For instance, a study in Brazil reported that 70% of 240 enterococci exhibited the *esp* gene, with *E. faecalis* and *E. faecium* accounting for 70.1% and 68.4% of those, respectively [27]. However, only 13 *E. faecium* isolates were enrolled in their study. In another study, 60% and 41.5% of clinical enterococci exhibited the *esp* gene in Puerto Rico and Southern California, respectively [40]. Prevalence rates of only 33.3% and 30% of the *esp* gene were detected in *E. faecalis* isolates in these two study locations. In contrast, higher detection rates of the *esp* gene (73% and 83%) were reported among *E. faecium* isolates in the same locations. A smaller number of *E. faecium* isolates were also observed in their study. Enterococcal surface protein (Esp) is an important virulence factor that is located on a pathogenicity island [41]. This potential surface protein is expressed on the cell wall of the enterococci and it is known to be predominantly found in nosocomial strains of *E. faecium*, which is in accordance with our findings [13]. Interestingly, the *esp* gene was more prevalent in clinical enterococci than environmental enterococci, as reported in a study in Australia [42]. In contrast to our findings, in a local study involving 90 *E. faecalis* and 12 *E. faecium* isolates from three different hospitals in Northeastern Malaysia, *E. faecalis* exhibited the *esp* gene more frequently than *E. faecium* (49.2% versus 30.8%, respectively) [43]. It seems that different hospital settings may produce different distributions of the *esp* gene among enterococci, even from the same geographic location. The possession of the *esp* gene in *E. faecalis* and *E. faecium* isolates has been implicated in chronic infections of the urinary system in animal models. It has been observed that Esp could increase the pathogenicity of *E. faecium* by adhering to the uroepithelial cells [44]. Similar observations have been reported in *E. faecalis* isolates as well [45]. Recent data have also shown that the induction of the expression of pro-inflammatory cytokines was initiated by *esp* via the activation of the NF-ĸB pathway in vitro [46]. However, the exact mechanism of the activation is still unknown.

Moreover, the genetic lineage of *E. faecium* is distinct from enterococcal gut flora [41]. Thus, it seems that nosocomial *E. faecium* strains have developed a special trait for their versatile adaptation to well-established hospital environments. This is further supported by the recognition of clade A or hospital-associated clade of nosocomial *E. faecium* isolates through the whole genome sequencing-based studies [47]. These strains are equipped with many virulence genes including *esp* and others. Surprisingly, the presence of the *esp* gene in *E. faecium* isolates has been shown to signify potential outbreaks in hospitals [37,41]. Regarding *E. faecalis*, there is no distinct genetic trait that has been reported among this species in the literature until now. This could be explained by the possibility of extensive gene recombination, which may have occurred since *E. faecalis* is ubiquitously found in almost all animals and insects which in turn could lead to the absence of predominant clones [47].

Since the *esp* gene is an important marker for pathogenicity island (PAI) and biofilm formation in clinical enterococci, it is worth examining the possible association between the presence of the *esp* gene and antimicrobial resistance. In our study, *esp*–positive enterococci were strongly associated with resistance to ampicillin (*p* = 0.032) and piperacillin/tazobactam (*p* = 0.012). Since the majority of the resistance was due to *E. faecium*, it can be concluded that the *esp*–positive *E. faecium* isolates were resistant to these antibiotics. Interestingly, hospital-adapted strains of *E. faecium* were strongly associated with ampicillin resistance and the acquisition of PAI, such as the *esp* gene and clonal-complex 17 (CC-17) [41]. More interestingly, *esp*–positive *E. faecium* isolates demonstrated higher resistance rates to β–lactams drugs in Sweden, which is consistent with our study [48]. It has been postulated that the absence of Esp—a large cell wall protein—would lead to subtle changes to the cell wall of enterococci, that could indirectly modify the susceptibility pattern toward ampicillin [49].

Enterococcal biofilm formation is a very complex process and the involvement of potential virulent factors in this process is highly contentious [35]. The *esp* gene has been shown to be important in biofilm formation [20]. The interaction between the *esp* gene and biofilms would establish the success rates of enterococcal infections, especially for those strains that are isolated from blood and urine samples [17,18]. In our study, there was no significant association between the *esp* gene (in both species) and biofilm formation (*p* = 0.934). Similar observations were also reported in several studies [50,51]. It has been concluded that the Esp protein is not crucial for the formation of biofilms and other biofilm-associated proteins—such as gelatinase (encoded by *gelE*) and aggregation substance (encoded by *agg*)—may be involved [13,49]. Nonetheless, a significant association between the *esp* gene and biofilm formation was observed among 240 clinical enterococci in a study in Brazil (*p* < 0.0001) [27].

Our study has several limitations. Firstly, the current study has only involved clinical samples from a hospital, thus, the findings cannot be generalized to all Malaysian hospitals. A multicenter study could produce more reliable information in future. Secondly, other samples from animals and the environment should be included to study the genetic evolution of enterococci, because any significant findings may be useful to relevant authorities in managing enterococcal diseases by a One Health Concept accordingly. Thirdly, it would be interesting to investigate the loci of β–lactams resistance and *esp* genes via molecular approaches. We could thereby ascertain whether the genes are carried by the same or different mobile genetic elements. Lastly, few other biofilm-related genes that are involved in enterococcal biofilm formation should be included as biofilm formation is a very complex process and multiple virulent genes may be involved. Nonetheless, our findings could offer an insight into the current status of clinical enterococci which is biologically and geographically different from other strains reported in many countries, and to our knowledge, this is the only study investigating the association between the *esp* gene with the susceptibility patterns of selected antibiotics and biofilm formation that has been reported so far in Malaysia.

## 5. Conclusions

In conclusion, our *E. faecium* isolates display higher resistance rates toward selected antibiotics (in particular of ampicillin and tazobactam/piperacillin) compared to *E. faecalis*, which could change the therapeutic options for patients infected with this strain. The presence of the *esp* gene among antibiotic-resistant *E. faecium* isolates would ensure their successful establishment in hospital settings, which may have implications for the management of enterococcal infections in hospitals. Hence, the close monitoring of ampicillin and tazobactam/piperacillin-resistant *E. faecium* isolates is crucial for the development of effective control measures in future.

## Figures and Tables

**Table 1:** Rates of antibiotic resistance in clinical enterococci.

Antibiotic	*Enterococcus faecalisn* %	*Enterococcus faeciumn* %
Penicillin	4 (7.7)	23 (82.1)
Ampicillin	1 (1.9)	26 (92.8)
Tazobactam/piperacillin	1 (1.9)	28 (100)
High-level gentamicin resistance (HLGR)	13 (25.0)	25 (89.3)
Vancomycin	0 (0)	0 (0)
Teicoplanin	0 (0)	0 (0)

**Table 2:** The association between antibiotic susceptibility patterns and the *esp* gene among clinical enterococci.

Antibiotic Susceptibility Patterns	Enterococcal Surface Protein (*esp*) Gene *n* (%)		*p* Value
	Positive	Negative	
PEN ^S^	29 (30.5)	24 (22.5)	0.481
PEN ^R^	17 (15.5)	10 (11.5)	
AMP ^S^	26 (30.5)	27 (22.5)	0.032 *
AMP ^R^	20 (15.5)	7 (11.5)	
HLGR ^S^	21 (24.2)	21 (17.9)	0.154
HLGR ^R^	25 (21.9)	13 (16.2)	
TZP ^S^	24 (29.3)	27 (21.7)	0.012 *
TZP ^R^	22 (16.7)	7 (12.3)	

PEN: penicillin, AMP: ampicillin, TZP: tazobactam/piperacillin, HLGR: high-level gentamicin resistance (gentamicin 120), S: sensitive, R: resistant. Vancomycin and teicoplanin were excluded for the analysis for all enterococci were sensitive to both antibiotics. * *p*-value < 0.05 was considered significant.

**Table 3:** The association between the *esp* gene and biofilm formation among clinical enterococci.

*esp* Gene	Biofilm Formation *n* ^†^ (%)		*p* Value *
	Yes	No	
Positive	24 (52.2)	22 (47.8)	0.934
Negative	17 (53.1)	15 (46.9)	

^†^ = the total sample was 78 as 2 isolates were lost during biofilm study. * *p*-value < 0.05 was considered significant.

## Supplementary Materials

The following are available online at https://www.mdpi.com/1660-4601/16/18/3439/s1, Figure S1: The classification of biofilm formation among *E. faecalis* and *E. faecium* isolates; Figure S2: Gel electrophoresis image of *esp* gene.
