# Supplementary material for: Antibiotic Susceptibility Patterns, Biofilm Formation and esp Gene among Clinical Enterococci: Is There Any Association?
Source: Int J Environ Res Public Health. 2019 Sep 17;16(18):3439. doi: 10.3390/ijerph16183439 (PMC6765802; doi:10.3390/ijerph16183439)
Supplement: Supplementary file 1 [file ijerph-16-03439-s001.pdf]

Supplementary Materials

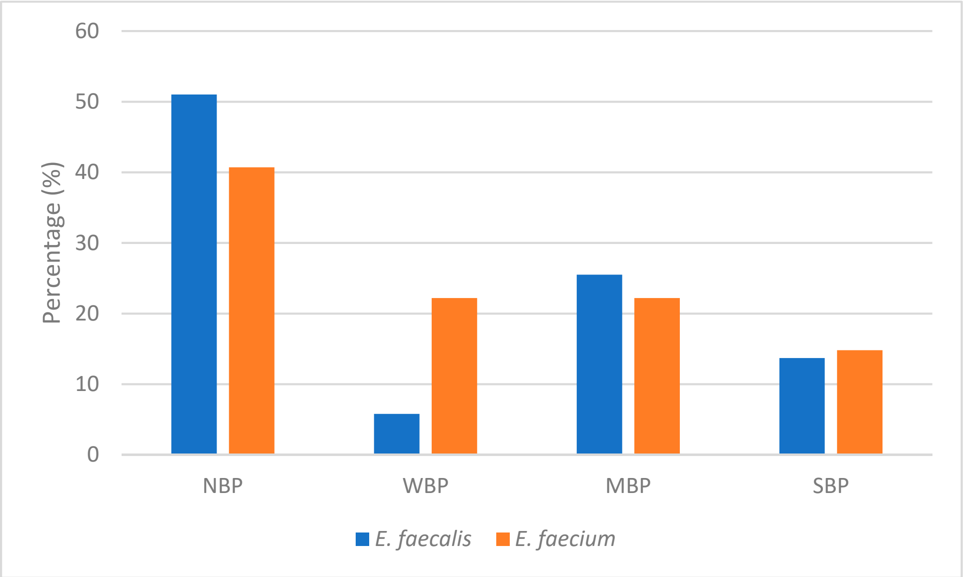

Figure S1. The classification of biofilm formation among *E. faecalis* and *E. faecium* isolates. NBP: no biofilm producer, WBP: weak biofilm producer, MBP: moderate biofilm producer, SBP: strong biofilm producer.

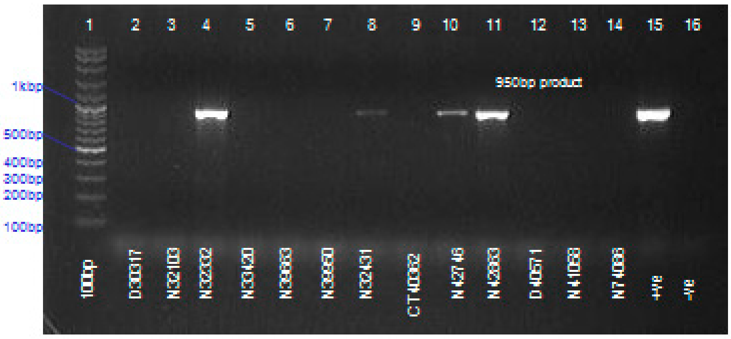

Figure S2. Gel electrophoresis image of *esp* gene (950bp). Lane 1: 100 base pair ladder (Vivantis). Lane 2-14: representative enterococci isolates with reference numbers. Lane 15: *Enterococcus faecalis* ATCC 29212 as positive control. Lane 16: water as negative control.
